# Impact Assessment of Gilgel Gibe Hydroelectric Dam on Schitosomiasis: A Cross Sectional Study in Southwest Ethiopia

**DOI:** 10.4314/ejhs.v20i2.69439

**Published:** 2010-07

**Authors:** Alemeshet Yami, Sileshi Kebede, Yoseph Mamo

**Affiliations:** 1 Department of Internal Medicine, Jimma University, email: alemyami@yahoo.com, corresponding author; 2 Department of Microbiology and Parasitology, Jimma University; 3 UCSD-ETHIOPIA, email: yosephm@ucsdeth.org

**Keywords:** Schistosomiasis, Gilgel-Gibe dam, Southwest Ethiopia

## Abstract

**Background:**

Intestinal schistosomiasis is prevalent in East Africa including Ethiopia. Constructed five years back, Gilgel Gibe dam is suspected to harbor the intermediate host for transmission of schistosomiasis. The objective of this study was to determine the prevalence of intestinal schistosomiasis and risk factors among school children.

**Methods:**

A comparative cross-sectional study was carried out in October 2008 in four Woredas bordering Gilgel Gibe dam, within 10 kilometers, and Bulbul, which is 30 Kms away from the dam. Children attending grades 1–8 in the schools located adjacent to the dam constituted the cases and those living in Bulbul constitute the controls. Using Epinfo version 6.0 for cross-sectional study, a sample size of 937 was determined. Sample size allocation was done 2:1 for cases and control. After interview, stool sample was collected and analyzed. Screening for the presence of intermediate host and physiochemical analyses of selected water bodies along the major water contact sites of the reservoir was also done Data were entered into computer and analyzed using SPSS for windows version 13.0.1.

**Results:**

Out of 624 sampled cases and 312 controls, 585 and 270 participated in the study giving a response rate of 93.8% and 86.5%, respectively. Four hundred seventy four (81.0% of the cases and 203 (75.2%) controls use latrine regularly. On stool examination, 406 (47.5%) children, 295 (50.4%) cases and 111 (41.1%) controls) were positive to intestinal parasites but only two children, both from the control groups, were positive for Schistosoma mansoni. The three river water samples on which malacological survey was done had similar physicochemical characteristics in many ways except high conductivity, pH and percent of dissolved oxygen concentration (milligram per liter) at one site where uninfected Biomphilaria Pfeifferi was found

**Conclusion:**

The study revealed that schistosomiasis is not yet a problem at Gilgel-Gibe dam. But, continuous surveying is required as the intermediate host is prevalent, the water bodies are suitable for the intermediate host and cases of schistosomiasis are identified 30 kms away the dam, in control area.

## Introduction

Schistosomiasis is a time proven indicator of public health impact of man made water reservoirs or irrigation schemes. In Africa and other parts of the tropical areas, the construction of dams mainly for irrigation and occasionally for large hydroelectric power projects has intensified community level infection by disease related to water and created new areas of transmission (1). Migration of workers from endemic areas to irrigation projects or major hydroelectric power plants, followed by contamination of reservoirs with human feces, and subsequent inevitable increasing contact of communities adjacent to the reservoir for the purpose of bathing, laundering in adults or swimming and playing in children will result in an expansion of infection in regions once free of infection (1,2).

Intestinal schistosomiasis is caused by *Schistosoma mansoni*; which is transmitted by fresh water snail *Biomphalaria Pfeifferi* or *Biomphalaria Sudanica*. The most important characteristics of water body that determine the habitation and density of the snail are physical factors including altitude, water temperature, turbidity, velocity and chemical factors like pH, anioin and cathioin concentrations, hardness and dissolved oxygen concentration (2,3). These factors should be within tolerable range of the intermediate host for effective transmission of the disease. Intestinal schistosomiasis is more prevalent in East Africa in contrast to the urinary schistosomiasis (2). In Ethiopia transmission of infection is mainly at an altitude of 1000–2000 meters above sea level (4). The disease was first described by Italian physicians in the northern part of the country as early as early as 1930's (5). From this classically endemic and occasionally isolated area, the disease has progressively spread to newer and more southern part of the country following the establishment of water resource development projects and migration of people from endemic areas to previously non endemic ones (6,7,8) hence the disease is now rapidly becoming an important public health threat.

In Jimma area, sporadic cases of schistosomiasis were seen and the intermediate host *Biomphalaria Pfeifferi* has also been identified from the major river crossing the town and also in a neighboring town of Agaro, 50 kilometers west (8). Gilgel Gibe dam, constructed five years back, is suspected to harbor the intermediate host for schistosomiasis. In addition to previous findings of the intermediate host in the region the altitude of the man-made lake and its tributary river and streams are in favor of this suspicion. Manisa and Lega-Gibe rivers are two tributaries of the Dam and measurement done during data collection showed the rivers are found at the height of 1720 meters and 1621 meters above sea level, respectively at crossing places for human and animals. Bulbul River has also similar topography with altitude of 1689 meters.

Due to the relative high cost of molluscicides, no public health intervention measure is in place in Ethiopia. Because of the toxicological clearance of Endod (*phylolacca dodecondra*), however, since 1991 interest has rekindled in the use of this local molluscicidal plant for community based intervention (9). Another aspect of intervention modality is intermittent mass treatment of school children with single dose of praziquantel; cutting the chain of transmission and perpetuation of infection in the community. This also has to go hand in hand with health education to decrease the risk of exposure and contaminative behavior but also provide safe water and adequate latrines (10).

The objective of the study was to determine the prevalence of intestinal schistosomiasis, and risk factors for infection among school children within. The other objectives of the study were to identify for the presence of intermediate host to *Schistosoma mansoni* along the major water contact sites of the reservoir and to determine the physiochemical characteristics of the water bodies. This would serve as a baseline for future monitoring of impact of the dam on possible spread of infection; warn for an early control interventions through environmental and mass treatment strategies, and to evaluate the impact of such public health interventions.

## Methods

A descriptive cross-sectional study was carried out in October 2008 in four Woredas of Jimma Zone bordering Gilgel Gibe Hydroelectric dam. The dam which started to collect water in 2001 and is 250 kms Southwest of Addis Ababa and 75 kms Northeast of Jimma City. It covers an area of 51 Km^2^ at an altitude of 1670 meters above sea level, and holding around 668 million mm^3^ of water (11). The four “Woredas” (districts) bordering the dam are Omonada, Sekoru, Tiroafeta and Kersa with 6, 4, 5, and 2 “Kebeles” (samllest administrative unit) within ten kilometers from the shoreline of the dam, respectively. There was one elementary school in each Kebele. The other study area was Bulbul, located in Kersa Woreda and 30 Kms away from the dam. Five years back 1100 households were resettled from the land submerged by the dam to this Woreda. There were two schools where the resettled children attend.

Children attending grades 1–8 in the schools located within 10 Kms from Gilgel Gibe dam and those living 30 Kms away from the shore line in the four bordering woredas were the study subjects. The inclusion criteria for the study were;

1. Children between the ages of 7–16, attending in a school within 10 Kms from the dam, or
2. Resettled children of age 7–16 years attending school in a village 30 km away from the dam.

These resettled children served as a control to the status before the dam was constructed

A total of 5,721 children within 10 Kms from the dam and 331 resettled children from the four Woredas bordering the dam fulfilled the inclusion criteria.

Sample size calculation was done using Epinfo version 6.0 for cross-sectional study. Proportion estimation was taken from previous study done in Finchaa (12). Accordingly, prevalence of schistosomiasis in the four woredas with in 10 Kms from the dam (P1) was assumed to be 40% and prevalence of schistosomiasis in Bulbul (30 Kms away from the shore) (P2) was assumed to be 30%. With 95% confidence level and 80% power, and adding 10% non-response rate, total sample size was 937. Because of the large population of school children in the schools around Gilgel Gibe shore and smaller re-settled children in the schools of Bulbul, sample size allocation; Gilgel Gibe: Bulbul (case to control) was made as 2:1. Therefore, 624 children selected from the schools around Gilgel Gibe dam comprised cases and 312 children from the schools in Bulbul comprised controls.

Out of 17 schools located around Gilgel Gibe dam, 4 were selected using multistage sampling technique and the two schools from Bulbul were the other study sites. Proportional allocation of samples to each school and classes with in a school was made and lottery method was used to select samples from each class. All the resettled children fulfilling the inclusion criteria in the two schools in Bulbul were included in the study.

Data on socio-demographic characteristics, risk behaviors like history of contact to river water and clinical symptoms were collected using a structured questionnaire prepared in Oromiffa. About 3 grams stool specimen was collected from each student using clean, dry, wide necked and leak proof plastic container. Upon delivery of the specimens the containers were labeled and each sample was emulsified in 5 to 10 ml of 10% formal saline solution and kept until examination.

Stool samples were transported to Jimma University Parasitology Department Laboratory for analysis within 2 hours of collection. A portion of stool sample from each subject was processed by formol-ether concentration technique, for the presence of *Schistosoma mansoni* ova and intensity (density) of infection per gram of stool ((Procedure is described in annex).

The altitude, longitude and latitude of the site and physicochemical characteristics of the water including PH, conductivity and dissolved oxygen concentration were done. In order to determine snail population density and S. *mansoni* infection rate, snails were collected from different sites. All accessible streams, swamps, boat boarding and disembarking banks, sites for laundering, bathing and swimming in the study area were surveyed for presence of snails which were then collected by scooping or forceps using plastic bags. The collected snails were kept in vials containing 2ml of water, transported to Jimma University Microbiology Department with in two hours and exposed for natural light to shed the cercaria. Snails were identified to species level using standard guidelines and keys (13).

Ethical clearance was obtained from Ethical Committee of Jimma University and permission was from the local administrators. Children who had intestinal parasites were treated single dose of Albendazole 400 mg and Praziquantel 40mg per Kg for cases of schistosomiasis.

## Results

Out of 624 sampled cases and 312 controls, 585 and 270 participated in the study giving a response rate of 93.8% and 86.5%, respectively. Of the 585 cases, 85 (14.5%) were from Degosso, 206 (35.2%) from Assendabo, 104 (17.8%) from Bore and 190 (32.5%) from Dimtu schools (Table 1).

**Table 1:** Distribution of study subjects by locality, Gilgel-Gibe dam, Southwest Ethiopia, 2008.

Location		Number	Percent
Controls (n =270)			
	Bulbul Main	88	32.6
	Bulbul Satellite	182	67.4
Cases (n = 585)			
	Dogosso	85	14.5
	Assendabo	206	35.2
	Bore	104	17.8
	Dimtu	190	32.7

Three hundred thirteen (53.5%) of cases and 141 (52.2%) of controls were females, 336 (57.4%) and 132 (48.9%) in the age group of 8–10 years, 511 (87.4%) and 258 (95.6%) Oromo, and 491 (83.9%) and 258 (95.6%) were Muslims, respectively. Four hundred seventy seven (81.5%) of the cases and 206 (77.0%) controls had latrine and 474 (81.0%) and 203 (75.2%) used latrine regularly. Five hundred twenty two (89.2%) and 248 (91.9%) of the cases and controls had river water contact. Cases and controls had statistically significant difference in age, ethnicity and religion (p < 0.05) where cases had larger proportion of young ages, other ethnic groups and Christian children; however, no difference was seen in sex distribution, availability and use of latrine and exposure to river water (Table 2).

**Table 2:** Baseline characteristics of the study participants, Gilgel-Gibe dam, Southwest Ethiopia, 2008

Variable			Cases (n=585)	Control (n=270)	Total (n=855)	P-value

			No(%)	No (%)	No (%)	
Sex						
	Male		272 (46.5)	129 (47.8)	401 (46.9)	
	Female		313 (53.5)	141 (52.2)	454 (53.1)	0.727
Age in years						
<8			51 (8.7)	24 (8.9)	75 (8.8)	
8–10			336 (57.4)	132 (48.9)	468 (54.7)	0.005
11–13			169 (28.9)	84 (31.1)	253 (29.6)	
≥ 14			29 (5.0)	30 (11.1)	59 (6.9)	
Ethnicity						
Oromo			511 (87.4)	258 (95.6)	769 (89.9)	
Amhara			25 (4.3)	10 (3.7)	35 (4.1)	0.000
Others§			49 (8.4)	2 (0.7)	51 (6.0)	
Religion						
		Muslim	491 (83.9)	258 (95.6)	749 (87.6)	0.000
		Christian	94 (16.1)	12 (4.4)	106 (12.4)	
Availability of latrine						
		Yes	477 (81.5)	206 (77.0)	685 (80.1)	0.125
		No	108 (18.5)	62 (23.0)	170 (19.9)	
Regular use of latrine						
	Yes		474 (81.0)	203 (75.2)	677 (79.2)	0.051
	No		111 (19.0)	67 (24.8)	178 (20.8)	
History of river water contact						
	Yes		522 (89.2)	248 (91.9)	770 (90.1)	
	No		63 (10.8)	22 (8.1)	85 (9.9)	0.234

§ Gurage, Tigre, Yem, Dawro

On stool examination, 406 (47.5%) children (295 (50.4%) cases and 111 (41.1%) controls) were positive to intestinal parasites but only two children (0.7%), both from the control group, were positive for Schistosoma (Table 3).

**Table 3:** Participants stool microscopic examination result by location; Gilgel-Gibe dam, Southwest Ethiopia, 2008

Stool Microscopy Result	Case (n=585) No (%)	Control (n=270) No (%)	Total No (% )
Positive for any intestinal parasite	295(50.4%)	111 (41.1%)	406 (47.5)
Positive for Schistosoma mansoni	0(0%)	2 (0.7%)	2(0.2%)
Negative for any intestinal parasite	290(49.6%)	159(58.9%)	449(52.5%)

The three river sites where snail collection was done had similar altitude, latitude and longitude. The physicochemical characteristic of the three river water was similar in many ways except that Lega Gibe river water had high conductivity (436), pH(8.23) and % DO2(7.2). *Biomphalaria Pfeifferi* was found only in one of the rivers (Lega-Gibe) feeding the dam. In Lega Gibe river forty fresh water snails of the species *Biomphalaria Pfeifferi* were collected and all were un-infected (Table 4).

**Table 4:** Malacological and environmental survey, Gilgel-Gibe dam, Southwest Ethiopia, 2008

Variable		Site 12 Gilgel-Gibe (Manisa river)	Site 11 Gilgel-Gibe (Lega-Gibe river)	Site 13 Bulbul (Bulbul river)
Physicochemical characteristics of water				
	Water T (^0^c)	18	18.2	18.3
	Ambient T (0c)	23	24	24
	Conductivity	90	436	70
	pH	7.22	8.23	7.24
	Turbidity(NTU)	15	28	35
	%DO2(mg/l)	6.28	7.2	6.28
	Velocity (m/s)	0.15	0.1	0.4
	Water depth(m)	3	0.2	0.6
	Water width(m)	0.1	4	6
	Color	no	no	no
	Smell	no	no	no
	Hardness as Caco_3_ mg/l)	15	52	20
	Chloride (mg/l)	13	2.49	2.99
Biomphilaria species				
	Availability	No	Yes	No
	Status of infection	Not applicable	Uninfected	Not applicable

Finally a univariate analysis was done to identify predictors of risk factors for schistosomiasis revealed that control children from Bulbul had no significant difference from cases at the Gilgel Gibe in terms of frequency of water contact. Similar analysis showed on both controls and cases that males had nearly two times more risk than females [OR: 1.913 (95% CI; 1.191, 3.072)] and Muslims three times more than Christians [OR: 0.35 (95% CI; 0.205, 0.599))] had river water contact. Age group 8–10 years, 11–13 and 14 and above had 2, 18 and 21 times more river contact than those below 8 years [OR 2.521 (95% CI;1.411, 4.502), 18.036 (95% CI; 6.487, 50.148), 21.091 (95% CI; 2.737, 162.520)], respectively. Being Amhara was with 25% less river water contact [OR 0.252 (95% CI; 0.130, 0.490). However, on multivariate analyses, only male sex [AOR 1.975 (95% CI; 1.205, 3.237)] and age groups 8 years or more remained to increase risk of river water contact [AOR 2.547 (95% CI; 1.395, 4.651), 17.648 (95% CI; 6.258, 49.774) and 21.112 (95% CI; 2.698, 165.197), respectively] (Table 5).

**Table 5:** Predictors for any river water contact; Jimma zone, 2008.

Variable		Contact to any river water No (%)	Crude Odds ratio (95% CI)	Adjusted Odds ratio (95% CI)
Sex				
	Male	373 (93.0%)	1.913 (1.191; 3.072)	1.975 (1.205; 3.237)
	Female	397 (87.4%)	1	1
Religion				
	Muslim	686 (91.6)	1	
	Christian	84 (79.2)	0.35 (0.205; 0.599)	
Age				
	<8	55 (73.3%)	1	1
	8–10	409 (87.4%)	2.521 (1.411; 4.502) 18.036	2.547 (1.395; 4.651)
	11–13	248 (98.0%)	(6.487; 50.148) 21.091	17.648 (6.258; 49.774)
	≥ 14	58 (98.3%)	(2.737;162.520)	21.112 (2.698; 165.197)
Ethnicity				
	Oromo	702(91.3)	1	
	Amhara	31(88.6)	0.252 (0.130; 0.490)	
	Other	37(72.5)		
Location				
	Bulbul	248(91.9%)	1	
	Gilgel-Gibe	522(89.2%)		

## Discussion

Previous studies revealed that children between ages 7–16 are most affected segment of the community because of increased exposure to water through playing and swimming, the disease becomes less prevalent beyond the age of 16 years as a result of immune control and death of the adult warm as the years go by (2). Schistosomiasis is found to cause stunting and decreased mental development in children. Heavier worm loads common in this age group make them an important element in transmission and hence strategically important for public health survey and intervention.

Studies conducted among school children in different parts of Ethiopia reported prevalence of intestinal schistosomiasis ranging from 30–70% (9, 14–20). The finding in the current survey is by far different from the above studies. This study demonstrated that there is no intestinal schistosomiasis in the four Woredas within 10 Kms from the shore line. The findings at Bulbul (a control site) of schistosoma infection is not surprising but has proved that the risk to schistosoma infection of those children who had been resettled to it from the dam area had not been smaller. The findings are not significant enough to claim that the Bulbul area is at greater risk of infection but strengthens the conclusion that at the moment the dam area and its surroundings had not put community at greater risk of infection.

One possible explanation could be that five years since construction may be a bit early to expect any impact of the dam on the spread of schistosomiasis and hence show significant changes in its prevalence in the area. This is consistent with the research finding done in central Côte d'Ivoire where the prevalence of S. mansoni did not change after thirteen years of dam construction (21), but the result is in contrary to research done in Senegal where an outbreak of S. mansoni was observed only 3 years after the Diama Dam became operational (22,23)

Though not significant but to the contrary those who resettled in Bulbul from these Woredas had intestinal schistosomiasis with prevalence rate of 0.7%; this may have been due to previous infection from Gilgel Gibe Dam area or recent one acquired at Bulbul. Therefore, if the infection is due to prior exposure to dam area then this again proves that the dam area currently doesn't pose greater danger in terms of risk of infection to schistosomiasis than it had five years earlier. On the other hand prior exposure doesn't seem to be realistic, because had this been the reason; another cases of Schistosomiasis could have been identified around the Gilgel gibe dam. If, however, the assumption is that infection has been acquired after resettlement we must then concur that disease risks should be made and necessary measures should be taken before choosing sites for re-settlers during construction of new dams.

According to the study done in Finchaa; prevalence of *Schistosoma mansoni* was 40% at the Finchaa Valley Elementary School located near the Finchaa Dam outlet and 29.5% among family members of laborers of the Finchaa State Farm located some 40 km downstream from the dam (12). Although the study is the only study so far in Ethiopia which helps to compare the prevalence of infection according to contact and distance from the dam, the study groups have different characteristics which would usually create bias and affect conclusions made. Our study has on the other hand focused on school children and their risk in terms of various sociodemographic variables and have shown that age ,sex, religion are few of the determinants of contact with river and Dam water contact.

Analysis of physiochemical characteristic of selected water bodies in the study area found that the values lie within the tolerable range of the snails as evidenced by other studies (3, 24,25)

The conclusion drown from this study is that there is no schistosomiasis in the woredas bordering Gilgel gibe dam and no immediate danger to the surrounding community, though we would like throw a word of caution as the presence of the intermediate host for intestinal schistosomiasis in the area proximal to the dam and suitability of water bodies for survival of the intermediate host indicates possible future risk for the introduction of the disease to this area. This together with a very high degree of risk behavior such as contact with potentially infective river or dam water and a significant proportion practicing outdoor defecation, one would anticipate a rapid spread of infection in the community once the chain reaction starts.

Therefore, we recommend conducting a regular malacological survey to monitor the degree of colonization of streams with the population of *Biomphalaria Pfeifferi* or *Biomphalaria Sudanica* and their infestation rate with *Schistosoma Mansoni*. This should go hand in hand with continuous survey of school children and treatment of infected cases. It may also be wise to initiate preventive activities such as promoting awareness on schistosomiasis to the communities surrounding the dam as well as small scale eradication of intermediate host from infested streams with the use of locally available natural molluscicides (Endod) and other novel strategies, hence also evaluating the utilization of this novel intervention (26, 27,28).
